# The classification capability of the Asia Pacific Colorectal Screening score in Korea: an analysis of the Cancer Screenee Cohort

**DOI:** 10.4178/epih.e2021069

**Published:** 2021-09-16

**Authors:** Xuan Quy Luu, Kyeongmin Lee, Jeongseon Kim, Dae Kyung Sohn, Aesun Shin, Kui Son Choi

**Affiliations:** 1 Department of Cancer Control and Population Health, Graduate School of Cancer Science and Policy, National Cancer Center, Goyang, Korea; 2 Department of Cancer Biomedical Science, Graduate School of Cancer Science and Policy, National Cancer Center, Goyang, Korea; 3 Center for Colorectal Cancer, Research Institute and Hospital, National Cancer Center, Goyang, Korea; 4 Department of Preventive Medicine, Seoul National University College of Medicine, Seoul, Korea

**Keywords:** Mass screening, Colorectal neoplasms, Risk assessment, Colonoscopy

## Abstract

**OBJECTIVES:**

This study aimed to validate a simple risk assessment tool for estimating the advanced colorectal neoplasia (ACN) risk at colonoscopy screenings and potential factors relevant for implementing this tool in the Korean population.

**METHODS:**

Our study analyzed data from the Cancer Screenee Cohort Study conducted by the National Cancer Center in Korea. The risk level was assessed using the Asia Pacific Colorectal Screening (APCS) score developed by the Asia-Pacific Working Group on Colorectal Cancer. Logistic regression models were used to examine the associations between colorectal-related outcomes and the risk level by APCS score. The discriminatory performance of the APCS score for various colorectal-related outcomes was assessed using C-statistics.

**RESULTS:**

In 12,520 individuals, 317 ACN cases and 4,528 adenoma cases were found. The APCS tool successfully classified the study population into different risk groups, and significant differences in the ACN rate and other outcomes were observed. The APCS score demonstrated acceptable discrimination capability with area under the curve values ranging from 0.62 to 0.65 for various outcomes. The results of the multivariate logistic regression model revealed that the high-risk group had a 3.1-fold higher risk of ACN (95% confidence interval, 2.08 to 4.67) than the average-risk group. Body mass index (BMI) was identified as a significant predictor of ACN in both multivariate and subgroup analyses.

**CONCLUSIONS:**

Our study highlighted significant differences in colorectal-related screening outcomes by colorectal risk level measured using the APCS score, and BMI could be used to improve the discriminatory capability of the APCS score.

## INTRODUCTION

Colorectal cancer (CRC) is one of the most common types of cancer globally, not only in Western countries, and by 2040 there will be an estimated 3 million new cases of CRC that cause more than 1.5 million deaths [1]. Early detection initiatives related to CRC have been undertaken since the 1930s, when the evidence and definition were still ambiguous [2]. In the 1990s and early 2000s, the results of many clinical trials showed evidence supporting the effectiveness of CRC screening [3-6], after which many countries, especially Western countries with a high burden of CRC, rapidly adopted CRC screening in their national cancer programs [7]. Most cancer screening guidelines and programs suggest screening strategies for average-risk populations [7-9]. As a result, more effective methods for screening strategies targeting individuals with a high-risk of cancer have been neglected [7-9]. The United States Multi-Society Task Force on Colorectal Cancer and the Asia Pacific Working Group on Colorectal Cancer Screening highly recommend colonoscopies to detect both CRC and adenomas in patients with a high-risk of CRC and neoplasm [10,11]. These patients can be identified via a simple and accurate risk assessment tool such as the Asia Pacific Colorectal Screening (APCS) score, which is a well-validated scoring set developed by the AsiaPacific Working Group on Colorectal Cancer with the aim of providing a simple and useful tool for assessing the risk of CRC in Asian populations [12].

In 2017, the Korea Cancer Registry reported 28,111 CRC cases (16,653 in male and 11,458 in female), which was only exceeded by the number of stomach cancer cases [13]. Currently, the Korea National Cancer Screening Program (NCSP) recommends an annual fecal immunological test (FIT) as the primary test for individuals aged 50 or older without any further specification based on CRC risk, and a colonoscopy or double-contrast barium enema test is only provided as a follow-up test for individuals with FIT-positive results. In addition to the NCSP, fecal occult blood tests and colonoscopies are also conducted in outpatient and private health assessment centers as options for opportunistic screening. In particular, the number of people who choose to undergo a colonoscopy is increasing significantly. According to a nationwide population-based study, the number of colonoscopies performed increased 8-fold over a 12-year period, and the annual colonoscopic polypectomy rate also significantly increased for patients of both sexes and across all age groups [14]. Consequently, this poses a high burden on the entire Korean healthcare system, and more people are exposed to harms as a result of colonoscopy, including the risk of bleeding and perforation. However, the participation rate of CRC screening has been found to be the lowest of all cancer types. The results of a National Cancer Screening Survey show that the colonoscopy screening rate had reached 45.4% in 2018, while the fecal-based screening rate was still around 25% to 30% [15]. One of the reasons for the increased use of colonoscopy may be related to the ability to detect and remove polyps during the procedure. Therefore, colonoscopy is recommended as the initial test for screening those in high-risk groups. It is suggested that a more detailed, risk-based approach could reduce the burden of colonoscopy on the healthcare system and increase its effectiveness, as a result of which high-risk individuals can receive the maximum benefits of the screening program and low-risk individuals are protected from the harms associated with colonoscopy [16]. Therefore, we conducted this study to assess the classification capability of a simple risk assessment tool and to examine potential ways of supplementing this tool for the purposes of CRC screening and early detection in Korean population.

## MATERIALS AND METHODS

### Study setting and participants

Our study obtained data from the Cancer Screenee Cohort Study conducted by the National Cancer Center (NCC) in Korea, which was a study of a large hospital-based cohort [17]. People who visited the Center for Cancer Prevention and Detection at the NCC for cancer screening were asked to participate in the cohort. From August 2002 to the end of July 2014, there were 41,105 screenees enrolled in the cohort who completed the questionnaire. Among them, 18,825 participants were screened for CRC with a colonoscopy as the primary test. Since the main focus of our study was CRC screening in conjunction with a simple risk classification tool, we excluded individuals younger than 40 years of age (n=3,042), individuals with a history of CRC, individuals for whom CRC was detected during a colonoscopy screening but who had another type of primary cancer (n=104), and individuals with poor bowel preparation or who still had fecal matter in parts of the colon (n=290). Patients with incomplete information related to any variable (n=2,869) were also excluded. Ultimately, the first-visit information of 12,520 male and female was included in our final analysis.

### Measurements

Participants who agreed to enroll were interviewed using a structured questionnaire to obtain socio-demographic information such as age, sex, education, and household income as well as family history of CRC, comorbidities, and health-related behavioral factors such as smoking status and drinking status. Body mass index (BMI) was calculated using weight and height, which were collected via a physical exam, using the formula: weight (kg)/[height (m)]^2^. The remaining information, including screening test results and biopsy results, were collected accordingly at the Center for Cancer Prevention and Detection, NCC.

The biopsy results of colonic polyps were provided in detail in the colonoscopy diagnosis, where the histological findings and the number and location of all polyps were noted by physicians based on the pathology report. Adenomatous polyps were considered the conventional subgroup of tubular, villous, and tubulovillous polyps, and non-neoplastic polyps were considered to represent all other types [18,19]. Advanced adenomas were defined as polyps with a size of ≥ 10 mm, villous/tubulovillous histology, or high-grade dysplasia [19].

Cancer information was obtained from screening results, and during the follow-up period, it was ascertained using data linked to the Korean National Cancer Incidence Database from the Korea Central Cancer Registry, in which CRC cancer cases are identified using the International Classification of Diseases for Oncology, Third Edition (C18.0, C18.1, C18.2, C18.3, C18.4, C18.5, C18.6, C18.7, C18.9, C19.9, C20.9). In addition, to address the issue of missed cases and interval cases in cancer screening, participants were followed until December 31, 2017 to detect new cancer cases. Advanced colorectal neoplasia (ACN) cases were defined as either invasive cancer cases or advanced adenoma cases.

### Colorectal risk assessment

We utilized the APCS score developed by the Asia-Pacific Working Group on Colorectal Cancer for assessing the risk level of our study population. The tool measures 4 main risk factors (age, sex, family history of CRC, and smoking status) for assessing a participant’s individual APCS score [12], as summarized in Table 1. In this study, we divided our study population into 3 groups (averagerisk: 0-1; moderate-risk: 2-3; high-risk: 4-7).

### Statistical analysis

Descriptive statistics were used to assess the baseline characteristics of study participants. For identifying the significance of differences between participants according to their characteristics, the chi-square test was used for categorical variables and the 2-sample t-test was used for comparing 2 mean values. To compare predictive performance, we assessed sensitivity, specificity, the positive predictive value, and the negative predictive value for various colorectal-related outcomes of the APCS score and current CRC guidelines.

Univariate and multivariate logistic regression models were used to assess the associations between screening-related outcomes and the risk level by APCS score as well as by other related factors [11,20,21]. The discriminatory performances of the APCS score and current CRC screening guidelines for various colorectal-related outcomes were assessed using C-statistics, which are identical to the area under the curve (AUC) for binary outcomes. A value of 0.5 reflects no discrimination power, and a value of 1 indicates perfect discrimination power [22]. All statistical analyses were performed using Stata version 15 (StataCorp., College Station, TX, USA).

### Ethics statement

All enrollees provided informed consent and were apprised of the purpose of the study and their rights as participants. The current study was approved by the Institutional Review Board of the National Cancer Center, Korea on July 2, 2020 (approval No. NCC2020-0186).

## RESULTS

### General characteristics of study participants

A total of 12,520 individuals were included in our analysis (Table 2), of whom 65.1% were male. About half of the study participants were 40 years to 50 years of age and had completed a university degree or higher. In total, 59.4% of our study population had a household income of more than Koren won (KRW) 4 million per month, and 5,227 participants (41.7%) had at least 1 chronic disease. A total of 749 participants (6.0%) had at least 1 first-degree relative with CRC. While 44.6% of males and females never smoked, about 70.9% of people reported that they were alcohol drinkers. A total of 8,115 screenees (64.8%) had a BMI of more than 23 kg/m^2^. As a result of assessing individuals’ risk levels based on the APCS score, 6,071 (48.5%) participants were classified as moderate-risk and 3,706 (29.6%) participants were classified as high-risk. The distribution of general characteristics was significantly different between the APCS risk groups.

### Colorectal-related outcomes and risk group according to the Asia-Pacific Colorectal Screening score

**Figure 1.**
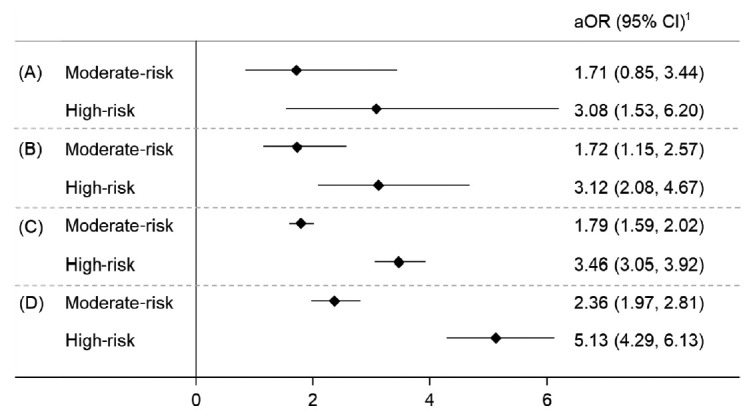
The odds ratios of having colorectal related outcomes (A) colorectal cancer, (B) advanced colorectal neoplasia, (C) adenoma, and (D) multipolyp. aOR, adjusted odd ratio; CI, confidence interval. ^1^Average risk as reference; adjusted for household income, com morbidity, alcohol drinking and body mass index.

Overall, the rates of invasive cancer and ACN were 0.9% and 2.5%, respectively (Table 3). At least 1 polyp was found in 44.1% of screenees, of whom about 67.2% had an adenomatous polyp. All of the rates were significantly higher for high-risk individuals than the lower-risk groups. The odds ratios (ORs) of having some colorectal-related outcomes by risk group are illustrated in Figure 1. High-risk participants were about 3.1 times more likely to have CRC than average-risk participants after adjusting for other factors. Similar results were also observed in the ORs for ACN (moderate-risk: OR, 1.72; 95% confidence interval [CI], 1.15 to 2.57; high-risk: OR, 3.12; 95% CI, 2.08 to 4.67). The ORs increased slightly when adenomatous polyps and multiple polyps were used as the outcome. The adjusted ORs of having adenoma were 1.79 (95% CI, 1.59 to 2.02) and 3.46 (95% CI, 3.05 to 3.92) for the averagerisk and high-risk groups, respectively.

**Figure 2.**
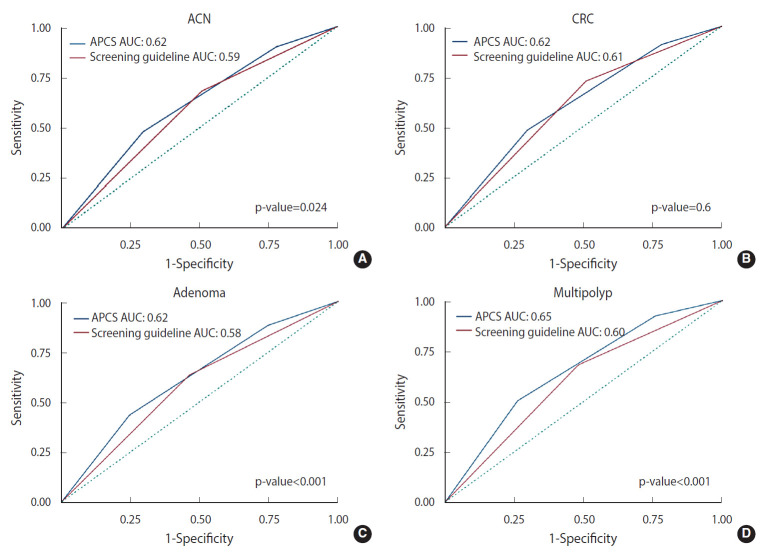
Area under the curve (AUC) curves for some colorectal related outcomes (A) advanced neoplasia (ACN), (B) colorectal cancer (CRC), (C) adenome, and (D) multipolyp by Asia-Pacific Colorectal Screening (APCS) score and screening guideline.

The discriminatory capability of the APCS score and current CRC screening guidelines was assessed using the AUC (Figure 2). While the classification capacity of the APCS score was similar for invasive cancer, ACN, and adenomatous polyps with an AUC value of 0.62, the AUC value for multiple polyps was 0.65. The AUC values of the APCS score were significantly higher than current screening guidelines for ACN, adenomatous polyps, and multiple polyps. When APCS results were organized to distinguish between high-risk individuals from other risk groups, the APCS score showed the highest sensitivity for multiple polyps (0.50; 95% CI, 0.48 to 0.52), followed by CRC (0.48; 95% CI, 0.39 to 0.58) and ACN (0.48; 95% CI, 0.42 to 0.54), whereas the specificity of the APCS score was above 0.70 for all outcomes (Table 4). In order to compare the APCS score to the current screening guidelines, APCS scores were further divided into 2 subgroups: moderate/high-risk, and average-risk. More than 90% of CRC and ACN cases were found in moderate/high-risk individuals. This figure was significantly higher than that of the classification system used by the current screening guidelines, which had a rate of 72.7% and 68.1% for CRC and ACN, respectively.

### Predictors of advanced colorectal neoplasia

In the univariate analysis, the average-risk group (OR, 1.93; 95% CI, 1.30 to 2.87), the high-risk group (OR, 3.61; 95% CI, 2.44 to 5.33), males (OR, 1.85; 95% CI, 1.42 to 2.41), the older age groups (50-59 years old: OR, 1.60; 95% CI, 1.22 to 2.09; ≥ 60 years old: OR, 3.19; 95% CI, 2.41 to 4.24), smokers and ex-smokers (OR, 1.67; 95% CI, 1.32 to 2.12), those with chronic diseases (OR, 1.36; 95% CI, 1.09 to 1.70), and those who were overweight (OR, 1.55; 95% CI, 1.20 to 1.99) showed positive associations with ACN. Accordingly, we ran 2 multivariate models for predictors of ACN. In model 1, besides risk level, BMI remained a significant predictor for ACN, and it was found that those with a BMI ≥ 23 kg/m^2^ had about a 32% increased risk of ACN after adjusting for the other factors. In contrast, a high household income was associated with a lower likelihood of ACN. In model 2, we inputted the original variables for risk calculation instead of the colorectal risk level. As a result, significant associations with ACN were observed between people aged 50-59 years old (adjusted odds ratio [aOR], 1.54; 95% CI, 1.17 to 2.02) and above 60 years old (aOR, 3.00; 95% CI, 2.20 to 4.08), current or ex-smokers (aOR, 1.42; 95% CI, 1.03 to 1.97) and those with a BMI ≥ 23 kg/m^2^ (aOR, 1.33; 95% CI, 1.03 to 1.73) (Table 5). In addition, the subgroup analyses for predictors of ACN by risk tiers are presented in Supplementary Material 1.

## DISCUSSION

In recent decades, many risk prediction models have been developed for colorectal-related outcomes with various sets of predictors [23]. Despite the sophistication and complexity of risk prediction models, a systematic review by Usher-Smith et al. [24] found that model performance did not significantly differ according to the level of model complexity in terms of variables. In addition, since the desired application for a risk prediction model is integration into general screening practices, a tool that is simple enough to be accessed easily by any medical professional is required for reducing the burden on the health system [25]. Therefore, the APCS score was identified as one of the best candidates for this approach, especially in an Asian context given that the tool was developed based on a cohort comprising individuals from 17 centers located in 11 Asian areas. It is recommended that each country should test the external validity of the tool and modify it accordingly for the best risk classification outcomes [10].

In the current study, the ACN detection rate and the adenoma detection rate (ADR) were 2.5% and 29.6%, respectively, and they were significantly lower for low-risk groups. Compared to the ACN rate in the original study on the development of the APCS tool (range, 3.0-4.5%) [12], our ACN rate was low. This difference could partially be explained by the characteristics of our cohort, in which half of the study population was younger than 50 years of age as opposed to the other study, in which only 34% of participants were under 50. Furthermore, a Korean multicenter study found an ACN rate of 2.2% [26], which was consistent with our study results. Though there is no single standard for ADR since large differences in this rate were observed between studies [27], the American Society for Gastrointestinal Endoscopy and American College of Gastroenterology Task Force on Quality in Endoscopy have suggested that this rate should be higher than 25% [27]. Given this suggestion, the ADR in our study was still above the recommended rate and was relatively similar to the results of the previous study from Korea (33%) [26].

In the natural history of CRC, more than 80% of CRC cases develop from adenomas [28]. The APCS tool was found to be useful for assessing various outcomes such as adenoma, advanced adenoma, multi-colonic polyps, and CRC. A previous study validated the APCS tool for predicting colonoscopy findings (polyps, adenoma, high-risk adenoma, and CRC) among an Australian study population [29]. Consequently, the removal of any type of CRC precancerous lesions via colonoscopy could eventually lead to a reduction in CRC incidence. Our study found significant differences in the prevalence of all colorectal-related outcomes. Compared to the average-risk group, the risk of those outcomes ranged from 1.72 times to 2.36 times higher for moderate-risk individuals and from 3.08 times to 5.13 times higher for high-risk individuals after adjusting for the other factors. Overall, the aORs for ACN were 1.72 (95% CI, 1.15 to 2.57) and 3.12 (95% CI, 2.08 to 4.67) for the average-risk group and high-risk group, respectively. Those ORs appear to be lower than another study that found that the high-risk and moderate-risk participants had a 4.3-fold and 2.6-fold increased likelihood of advanced neoplasia, respectively, compared to the lowest-risk group [12] and a study from China that found a 6.3-fold increase for high-risk individuals and a 3.3-fold increase for moderate-risk individuals [30].

Additionally, the AUC values of the APCS scores were 0.62 for CRC, ACN, and adenoma, and 0.65 for multiple polyps. This figure for ACN was slightly below the reported AUC value of the original study (0.64) as well as the pooled AUC value (0.63) found in a meta-analysis of 9 validation studies with about 93,000 participants [31]. The reported AUC values were not high compared to other risk prediction models for CRC, especially complex models with detailed clinical and genetic information. In a systematic review by Usher-Smith et al. [24] on prediction models for CRC, the discrimination of 37 models varied broadly from as low as 0.57 to as high as 0.88, and the models with the top discrimination included genetic and biomarker variables. Of the 19 models that only included information from a self-reported questionnaire, most had AUC values ranging from 0.6 to 0.7 except for the studies that used bootstrapping [24]. A risk-stratified approach in screening requires a tool that is simple and has a small number of components that can be used conveniently by any medical professional. As a result, the trade-off between simplicity and accuracy of the model is understandable, and simpler models are associated with lower accuracy. As a rule, AUC values of > 0.7 are generally considered to be good [22]. Nevertheless, with regard to a potential risk prediction tool tailored to screening recommendations for patients, Wells et al. [32] argued that any model with a C-statistic value of >0.5 might have some clinical utility, and a cross-validated discrimination of about 0.68 was considered to indicate good accuracy. Similarly, Wong et al. [33] also considered a C-statistic value between 0.6 and 0.7 to be acceptable with some clinical significance. Above all, our study results suggest that the APCS tool could perform well with the acceptable discriminatory capability not only for CRC or ACN, but also for other potential outcomes affecting Korean screenees, including adenoma and multiple polyps.

The acceptable discriminatory capability of this score in our study might suggest a promising approach in which high-risk individuals are directly screened via a colonoscopy to detect both cancer and precancerous lesions while low-risk individuals could still undergo a less invasive test for screening. This strategy was tested and demonstrated in a multicenter prospective study that found that 70% of cases of advanced adenomas were detected during screening, while 95% of cases of CRC were detected [34]. The effectiveness of this strategy was comparable to a strategy involving colonoscopy alone [35], but led to a substantially lower number of colonoscopy procedures in addition to reducing the substantial cost of the procedure for healthcare systems, even in developed countries. In addition, the personalization of colorectal risk could potentially improve the health-consciousness and knowledge of individuals [36] and create opportunities to cultivate healthy behaviors, including individuals’ intentions to participate in cancer screening programs as recommended [37]. Consequently, this strategy is expected to increase the participation rate of CRC screening in Korea, which was found to be lower than the rate of screening for other cancer types included in the Korea NCSP [38].

This study had several limitations that must be considered. First, due to a lack of information, some colonoscopy-related indicators (adverse events, endoscopist experience, history of colonoscopy) and dietary information were not included in the analysis. In addition, since the NCC is the largest authorized center in Korea for cancer screening, there may be fewer concerns regarding the process of diagnosis and treatment, endoscopist performance, and the quality of colonoscopy than at smaller centers. Regarding the history of colonoscopy and colorectal-related issues, we analyzed only the first visit of asymptomatic participants to minimize possible effects on our study results. Future studies should carefully consider the above information. Second, since the participants comprised a cancer screenee cohort and underwent opportunistic colonoscopy-based CRC screening, there was a possibility of self-selection bias in which cancer screening participants were likely to already have a high socioeconomic status and a high degree of health consciousness. Lastly, the results were based on a single-center cohort and could be limited in terms of their generalizability to the Korean population at large. Despite these limitations, the screening population for whom data were linked to several sources, including cancer registry data and pathology data, enabled us to directly investigate cancer screening-related issues, especially colonoscopy-based colorectal screening, to identify the most appropriate method for conducting cancer screening in Korea.

In conclusion, our study highlighted that the APCS score could successfully classify Korean screenees into different risk groups with acceptable discriminatory capability. In the context of fecal-based screening tests being providing universally for screening for an average-risk population, our results provide insight into a simple risk assessment tool that can be used for improving the effectiveness of screening programs without causing a heavy burden on the healthcare system and the national budget. Furthermore, our study results also suggest that BMI is a significant predictor of colorectal-related health outcomes, which could be potentially added to the original APCS score for improving its discriminatory power. A future study on attitudes among the general population and health professionals toward a risk-based screening approach that uses a simple risk assessment tool should be conducted.

## Figures and Tables

**Table 1.** Asia-Pacific Colorectal Screening score categories

Risk factor	Criteria	Points
Age (yr)	<50	0
	50-69	2
	≥70	3
Sex	Female	0
	Male	1
Family history of colorectal cancer (first degree)	Yes	0
	No	2
Smoking	Non-smoker	0
	Current or ex-smoker	1

**Table 2.** Baseline characteristics of the study population

Characteristics	Total (n=12,520)	Colorectal risk level^1^			p-value^2^
		Average (n=2,687)	Moderate (n=6,071)	High (n=3,762)	
Sex					<0.001
Male	8,154 (65.1)	2,084 (77.6)	2,160 (35.6)	122 (3.2)	
Female	4,366 (34.9)	603 (22.4)	3,911 (64.4)	3,640 (96.8)	
Age (yr)					<0.001
<50	6,095 (48.7)	2,687 (100)	3,212 (52.9)	196 (5.2)	
50-59	4,505 (36.0)	0 (0.0)	1,985 (32.7)	2,520 (67)	
≥60	1,920 (15.3)	0 (0.0)	874 (14.4)	1,046 (27.8)	<0.001
Mean±SD	50.94±7.49	44.59±2.80	50.48±7.40	56.21±6.10	
Education level					<0.001
Primary school or lower	903 (7.2)	63 (2.3)	551 (9.1)	289 (7.7)	
Middle/high school	5,284 (42.2)	1,240 (46.2)	2,499 (41.2)	1,545 (41.1)	
University or higher	6,333 (50.6)	1,384 (51.5)	3,021 (49.8)	1,928 (51.3)	
Household monthly income (10^6^ Korean won)					<0.001
<2.00	1,560 (12.5)	198 (7.4)	813 (13.4)	549 (14.6)	
2.00-3.99	3,521 (28.1)	696 (25.9)	1,781 (29.3)	1,044 (27.8)	
≥4.00	7,439 (59.4)	1,793 (66.7)	3,477 (57.3)	2,169 (57.7)	
Comorbidity					<0.001
No	7,293 (58.2)	2,101 (78.2)	3,434 (56.6)	1,758 (46.7)	
Yes	5,227(41.7)	586 (21.8)	2,637 (43.4)	2,004 (53.3)	
First-degree family history of CRC					<0.001
No	11,771 (94.0)	2,687 (100)	5,899 (97.2)	3,185 (84.7)	
Yes	749 (6.0)	0 (0.0)	172 (2.8)	577 (15.3)	
Smoking status					<0.001
Non-smoker	5,588 (44.6)	2,481 (92.3)	2,901 (47.8)	206 (5.5)	
Ex-smoker	1,641(13.1)	28 (1.0)	651 (10.7)	962 (25.6)	
Smoker	5,291 (42.3)	178 (6.6)	2,519 (41.5)	2,594 (69.0)	
Alcohol drinking					<0.001
Non-drinker/ex-drinker	3,639 (29.1)	1,029 (38.3)	1,899 (31.3)	711 (18.9)	
Drinker	8,881(70.9)	1,658 (61.7)	4,172 (68.7)	3,051 (81.1)	
BMI (kg/m^2^)					<0.001
<23	4,405 (35.2)	1,473 (54.8)	1,916 (31.6)	1,016 (27.0)	
≥23	8,115 (64.8)	1,214 (45.2)	4,155 (68.4)	2,746 (73.0)	

Values are presented as number (%). SD, standard deviation; CRC, colorectal cancer; BMI, body mass index.

1 Average-risk: 0-1; moderate-risk: 2-3; high-risk: 4-7.

2 The chi-square test was used for categorical variables, and analysis of variance was used for comparing means.

**Table 3.** Colorectal related outcomes by Asia-Pacific Colorectal Screening score risk tiers

Variables	Total (n=12,520)	Colorectal risk level^1^			p-value
		Average (n=2,687)	Moderate (n=6,071)	High (n=3,760)	
Colorectal cancer					<0.001
No	12,410 (99.1)	2,677 (99.6)	6,024 (99.2)	3,709 (98.6)	
Yes	110 (0.9)	10 (0.4)	47 (0.8)	53 (1.4)	
Advanced colorectal neoplasia^2^					<0.001
No	12,203 (97.5)	2,656 (98.8)	5,937 (97.8)	3,610 (96.0)	
Yes	317 (2.5)	31 (1.1)	134 (2.2)	152 (4.0)	
No. of polyps					<0.001
0	7,003 (55.9)	1,952 (72.6)	3,495 (57.6)	1,556 (41.4)	
1	3,419 (27.3)	573 (21.3)	1,693 (27.9)	1,153 (30.6)	
≥2	2,098 (16.8)	162 (6.0)	883 (14.5)	1,053 (28.0)	
Adenoma					<0.001
No	8,814 (70.4)	2,243 (83.5)	4,413 (72.7)	2,158 (57.4)	
Yes	3,706 (29.6)	444 (16.5)	1,658 (27.3)	1,604 (42.6)	

Values are presented as number (%).

1 Average-risk: 0-1; moderate-risk: 2-3; high-risk: 4-7.

2 Advanced colorectal neoplasia cases were defined as either invasive cancer or advanced adenomas.

**Table 4.** Performance characteristics of the APCS score and current screening guideline

Characteristics	Sensitivity (95% CI)	Specificity (95% CI)	PPV (95% CI)	NPV (95% CI)
Colorectal cancer				
APCS^1^	0.48 (0.39, 0.58)	0.70 (0.69, 0.71)	0.01 (0.01, 0.02)	0.99 (0.99, 0.99)
APCS^2^	0.91 (0.85, 0.96)	0.22 (0.21, 0.22)	0.01 (0.01, 0.01)	1.00 (0.99, 1.00)
Screening guideline^3^	0.73 (0.64, 0.81)	0.49 (0.48, 0.50)	0.01 (0.01, 0.01)	0.99 (0.99, 1.00)
ACN				
APCS^1^	0.48 (0.42, 0.54)	0.70 (0.70, 0.71)	0.04 (0.03, 0.05)	0.98 (0.98, 0.98)
APCS^2^	0.90 (0.87, 0.93)	0.22 (0.21, 0.22)	0.03 (0.03, 0.03)	0.99 (0.98, 0.99)
Screening guideline^3^	0.68 (0.63, 0.73)	0.49 (0.48, 0.50)	0.03 (0.03, 0.04)	0.98 (0.98, 0.99)
Adenoma				
APCS^1^	0.43 (0.42, 0.45)	0.75 (0.75, 0.76)	0.43 (0.41, 0.44)	0.76 (0.75, 0.77)
APCS^2^	0.88 (0.87, 0.89)	0.25 (0.24, 0.26)	0.33 (0.32, 0.34)	0.83 (0.82, 0.85)
Screening guideline^3^	0.63 (0.62, 0.65)	0.54 (0.53, 0.55)	0.36 (0.35, 0.38)	0.78 (0.77, 0.79)
Multiple polyps				
APCS^1^	0.50 (0.48, 0.52)	0.74 (0.73, 0.75)	0.72 (0.71, 0.73)	0.88 (0.87, 0.89)
APCS^2^	0.92 (0.91, 0.93)	0.24 (0.23, 0.25)	0.20 (0.19, 0.20)	0.94 (0.93, 0.95)
Screening guideline^3^	0.68 (0.66, 0.70)	0.52 (0.51, 0.53)	0.22 (0.21, 0.23)	0.89 (0.88, 0.90)

APCS, Asia-Pacific Colorectal Screening; ACN, advanced neoplasia; CI, confidence interval; PPV, positive predictive value; NPV, negative predictive value.

1 High-risk versus average/moderate-risk.

2 High/moderate-risk versus average-risk.

3 Aged 50 or older versus less than 50 years of age.

**Table 5.** Factors associated with ACN among the study population

Variables	Had ACN, n (%)		Univariate OR (95% CI)	Multivariate	
	No (n=12,203)	Yes (n=317)		Model 1 aOR (95% CI)	Model 2 aOR (95% CI)
Colorectal risk level^1^					
Average	2,656 (21.8)	31 (9.8)	1.00 (reference)	1.00 (reference)	-
Moderate	5,937 (48.6)	134 (42.3)	1.93 (1.30, 2.87)	1.72 (1.15, 2.57)	-
High	3,610 (29.6)	152 (47.9)	3.61 (2.44, 5.33)	3.12 (2.08, 4.67)	-
Sex					
Female	4,294 (35.2)	72 (22.7)	1.00 (reference)	-	1.00 (reference)
Male	7,909 (64.8)	245 (77.3)	1.85 (1.42, 2.41)	-	1.30 (0.89, 1.90)
Age (yr)					
<50	5,994 (49.1)	101 (31.9)	1.00 (reference)	1.00 (reference)	
50-59	4,387 (35.9)	118 (37.2)	1.60 (1.22, 2.09)	-	1.54 (1.17, 2.02)
≥60	1,822 (14.9)	98 (30.9)	3.19 (2.41, 4.24)	-	3.00 (2.20, 4.08)
First-degree family history of CRC					
No	11,479 (94.1)	292 (92.1)	1.00 (reference)	-	1.00 (reference)
Yes	724 (5.9)	25 (7.9)	1.36 (0.90, 2.06)	-	1.41 (0.93, 2.14)
Smoking status					
Non-smoker	5,484 (44.9)	104 (32.8)	1.00 (reference)	-	1.00 (reference)
Ex-smoker/smoker	6,719 (55.1)	213 (67.2)	1.67 (1.32, 2.12)	-	1.42 (1.03, 1.97)
Household monthly income (10^6^ Korean won)					
<2.00	1,499 (12.3)	61 (19.2)	1.00 (reference)	1.00 (reference)	1.00 (reference)
2.00-3.99	3,438 (28.2)	83 (26.2)	0.59 (0.42, 0.83)	0.63 (0.45, 0.88)	0.71 (0.50, 1.01)
≥4.00	7,266 (59.5)	173 (54.6)	0.59 (0.43, 0.79)	0.63 (0.47, 0.85)	0.75 (0.55, 1.03)
Comorbidity					
No	7,132 (58.4)	161 (50.8)	1.00 (reference)	1.00 (reference)	1.00 (reference)
Yes	5,071 (41.6)	156 (49.2)	1.36 (1.09, 1.70)	1.11 (0.88, 1.39)	1.04 (0.82, 1.31)
Alcohol drinking					
Non-drinker/ex-drinker	3,557 (29.1)	82 (25.9)	1.00 (reference)	1.00 (reference)	1.00 (reference)
Drinker	8,646 (70.8)	235 (74.1)	1.18 (0.91, 1.52)	1.03 (0.79, 1.34)	1.06 (0.80, 1.41)
BMI (kg/m^2^)					
<23	4,322 (35.4)	83 (26.2)	1.00 (reference)	1.00 (reference)	1.00 (reference)
≥23	7,881 (64.6)	234 (73.8)	1.55 (1.20, 1.99)	1.32 (1.02, 1.67)	1.33 (1.03, 1.73)

ACN, advanced colorectal neoplasia; CRC, colorectal cancer; BMI, body mass index OR, odd ratio; aOR, adjusted odd ratio; CI, confidence interval.

1 Average-risk: 0-1; moderate-risk: 2-3; high-risk: 4-7.

## SUPPLEMENTARY MATERIALS

Supplementary Material 1. Subgroup analysis by colorectal risk level for ACN related factors
