# Supplementary material for: The classification capability of the Asia Pacific Colorectal Screening score in Korea: an analysis of the Cancer Screenee Cohort
Source: Epidemiol Health. 2021 Sep 16;43:e2021069. doi: 10.4178/epih.e2021069 (PMC8654505; doi:10.4178/epih.e2021069)
Supplement: Supplementary Material 1. — Subgroup analysis by colorectal risk level for ACN related factors [file epih-43-e2021069-suppl.docx]

|  | Colorectal risk level^1^ | | |
| --- | --- | --- | --- |
| Variable | Average | Moderate | High |
|  | aOR (95% CI) | aOR (95% CI) | aOR (95% CI) |
| Household monthly income (10^6^ Korean won) | |  |  |
| <2.00 | 1.00 | 1.00 | 1.00 |
| 2.00-3.99 | 0.73 (0.19-2.77) | 0.76 (0.44-1.31) | 0.57 (0.36-0.90) |
| >= 4.00 | 0.69 (0.20-2.35) | 0.90 (0.55-1.47) | 0.49 (0.33-0.75) |
| Comorbidity |  |  |  |
| No | 1.00 | 1.00 | 1.00 |
| Yes | 1.43 (0.62-3.3) | 1.40 (0.99-1.98) | 0.87 (0.62-1.20) |
| Alcohol drinking |  |  |  |
| Non-drinker /ex-drinker | 1.00 | 1.00 | 1.00 |
| Drinker | 1.01 (0.49-2.1) | 0.72 (0.5-1.04) | 1.61 (1.00-2.61) |
| BMI (kg/m^2^) |  |  |  |
| <23 | 1.00 | 1.00 | 1.00 |
| >=23 | 0.91 (0.58-1.61) | 1.79 (1.16-2.76) | 1.24 (0.84-1.82) |
| ACN, advanced colorectal neoplasia; aOR, adjusted odd ratio; CI, confidence interval.  ^1^Average-risk: 0-1; moderate-risk: 2-3; high-risk: 4-7 | | | |

Supplementary Material 1: Subgroup analysis by colorectal risk level for ACN related factors
